# Involvement of Salicylic Acid and Other Phenolic Compounds in Light-Dependent Cold Acclimation in Maize

**DOI:** 10.3390/ijms21061942

**Published:** 2020-03-12

**Authors:** Magda Pál, Tibor Janda, Imre Majláth, Gabriella Szalai

**Affiliations:** Agricultural Institute, Centre for Agricultural Research, 2462 Martonvásár, Brunszvik 2, Hungary; pal.magda@agrar.mta.hu (M.P.); janda.tibor@agrar.mta.hu (T.J.); majlath.imre@agrar.mta.hu (I.M.)

**Keywords:** acclimation, antioxidants, chilling, phenylpropanoid pathway, salicylic acid, *Zea mays*

## Abstract

The exposure of plants to non-lethal low temperatures may increase their tolerance to a subsequent severe chilling stress. To some extent, this is also true for cold-sensitive species, including maize. In the present work, based on our previous microarray experiment, the differentially expressed genes with phenylpropanoid pathways in the focus were further investigated in relation to changes in certain phenolic compounds and other plant growth regulators. Phenylalanine ammonia lyase (PAL) was mainly activated under limited light conditions. However, light-induced anthocyanin accumulation occurred both in the leaves and roots. Chilling stress induced the accumulation of salicylic acid (SA), but this accumulation was moderated in the cold-acclimated plants. Acclimation also reduced the accumulation of jasmonic acid (JA) in the leaves, which was rather induced in the roots. The level of abscisic acid (ABA) is mainly related to the level of the stress, and less indicated the level of the acclimation. The highest glutathione (GSH) amount was observed during the recovery period in the leaves of plants that were cold acclimated at growth light, while their precursors started to accumulate GSH even during the chilling. In conclusion, different light conditions during the cold acclimation period differentially affected certain stress-related mechanisms in young maize plants and changes were also light-dependent in the root, not only in the leaves.

## 1. Introduction

Due to its subtropical origin, chilling is one of the most important factors limiting the spread and production of maize plants. Long-term exposure to temperatures around 10–15 °C may already decrease the capacity for biomass production. Lower chilling temperatures (0–5 °C) may lead to severe irreversible damage and the death of the plants [1,2]. Especially at continental climates, chilling tolerance at early stages of growth is a critical part of resistance to low temperature stress in maize plants.

Exposure of plants to low, but non-lethal, acclimating temperatures may increase their tolerance to a subsequent severe chilling stress [3,4,5]. To some extent, this is also true for cold-sensitive species, including maize. Better understanding the mechanisms that play a role in cold acclimation processes may help us to develop crop plants with higher levels of cold tolerance. It has been known for a long time that, without enough light during the cold hardening period, winter cereals—even winter cereals with a potentially high level of frost hardiness—are incapable of achieving a high level of freezing tolerance [6,7]. Acclimation to low temperatures also responds to light and temperature signals [8,9]. Light has been shown to mediate the development of freezing tolerance via several biological processes. These include photosynthesis-related processes, the expression level of stress-related genes and the synthesis of various protective compounds [10]. In the case of chilling sensitive plants, light during the cold period is mainly known as an extra stress factor inducing photoinhibition of photosynthesis [11]. As a consequence, photoinhibition may also contribute to the development of the chilling injury. However, we have recently shown that, in spite of the photoinhibitory effects, light during the cold acclimation period could also enhance the effectiveness of acclimation processes in young maize plants [12]. Similarly, moderate photoinhibition could also protect Photosystem I from photodamage under low temperature conditions in tobacco plants [13]. Furthermore, photoinhition of Photosystem I in *Arabidopsis* also protected the chloroplasts from oxidative damage [14].

It seems that light is at least as important factor as the temperature during the cold acclimation period. During the exposure of maize plants to cold acclimating temperatures, light influenced various light-related cold acclimation processes not only directly, but also at the gene expression and metabolomics levels. A microarray study showed that complex regulation mechanisms and interactions between cold and light signaling processes exist during the acclimation period. Numerous significantly differentially expressed genes that are involved in most of the assimilation and metabolic pathways were detected [12]. However, the exact mechanisms regarding how light may regulate the cold acclimation processes are still poorly understood.

Plants often react to biotic or abiotic stresses with an increase in the secondary metabolite levels. In relation to this, the increased activity of phenylalanine ammonia lyase (PAL) and other related enzymes can be observed. Phenolic compounds are naturally occurring substances in plants, and many of them play important roles in defence mechanisms and the scavenging of oxidizing molecules [15]. Salicylic acid (SA) is also a phenolic compound, and it plays an important signalling role in plants in various abiotic and biotic stresses [16,17]. Among other effects, SA may also induce the production of plant defensive metabolites, including other phenolic compounds and antioxidant systems [18,19].

In the present work, based on our previous microarray experiment [12], we further analysed the differentially expressed genes, focusing on the phenylpropanoid pathways. Furthermore, the changes in certain phenolic compounds, and other plant growth regulators, such as plant hormones and thiol compounds were analysed, in order to reveal their possible role in light-regulating signalling during the cold acclimation processes in young maize plants.

## 2. Results

### 2.1. Plant Hormones

**Figure 1:**
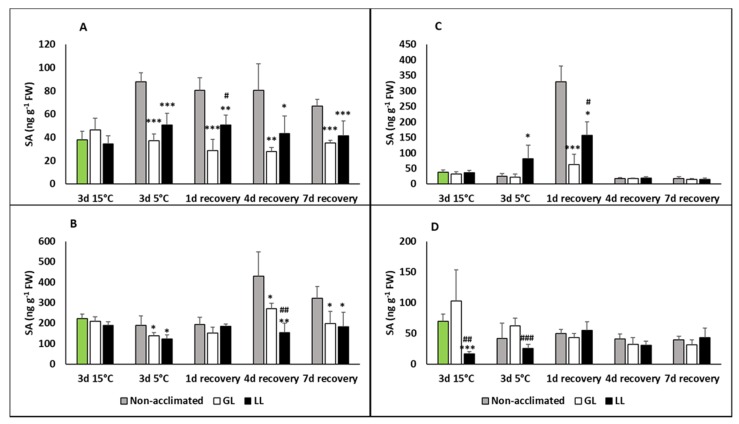
Changes in the salicylic acid (SA) contents during cold acclimation (15/13 °C), chilling (5 °C) and recovery in the leaves and roots of young maize plans: control plants (22/20 °C, 387 µmol m^−2^ s^−1^). Light intensities during hardening: growth light (GL): 387 µmol m^−2^ s^−1^; low light (LL): 107 µmol m^−2^ s^−1^. *, **, *** significant differences compared to the control plants on the same day at the *p* < 0.05, 0.01 and 0.001 levels, respectively. **^#^**, **^##^**, **^###^** significant differences compared to the GL plants on the same day at the *p* < 0.05, 0.01 and 0.001 levels, respectively. (**A**: free SA in the leaves; **B**: bound SA in the leaves; **C**: free SA in the roots; **D**: bound SA in the roots).

Growing young maize plants at cold acclimating temperatures (15 °C) did not cause significant changes in the free SA contents in the leaves compared to the control plants (Figure 1A). However, SA increased in a high manner during chilling at 5 °C in the non-acclimated plants, and it remained at this high level during the recovery period. Plants acclimated at low light (LL) had slightly higher free SA levels during recovery than the cold-acclimated one at growth light (GL). The bound SA level elevated only during recovery and only in non-acclimated plants (Figure 1B). While the bound SA was usually higher than the free SA in the leaves, this difference was less pronounced in the roots (Figure 1C,D). However, a substantial increase in the SA level could be detected in the free SA in the roots after a one-day recovery period. After a longer recovery, of 4 days or more, only low SA levels could be detected. The bound SA levels after the acclimation and chilling periods were significantly lower in the LL plants than in the control.

**Figure 2:**
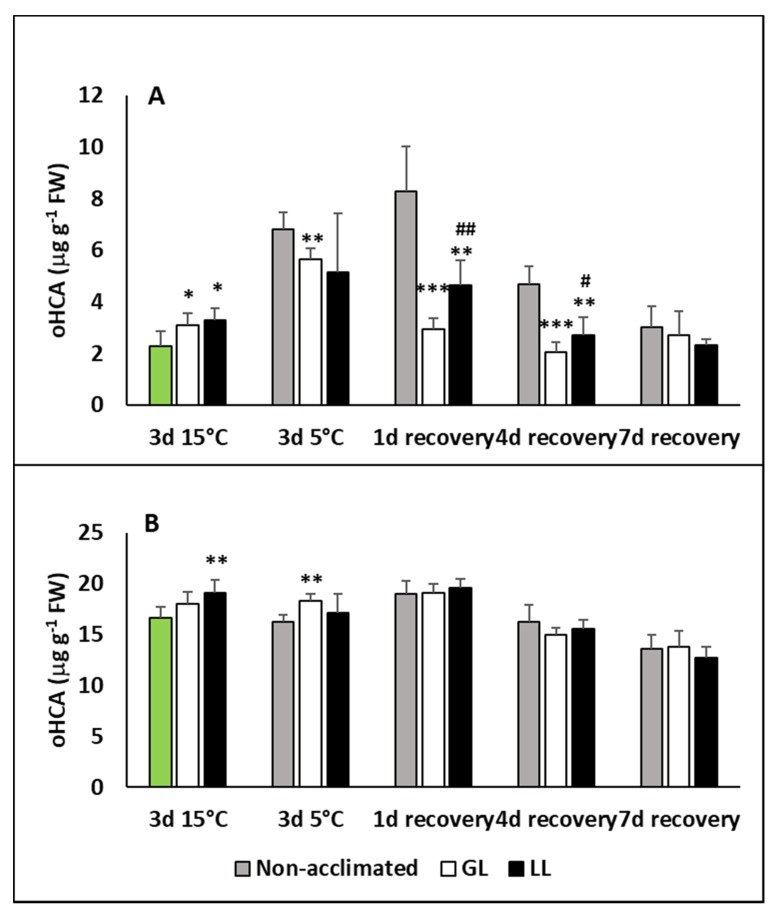
Changes in the ortho-hydroxycinnamic acid (oHCA) contents during cold acclimation (15/13 °C), chilling (5 °C) and recovery in the leaves and roots of young maize plants: control plants (22/20 °C, 387 µmol m^−2^ s^−1^). Light intensities during hardening: GL: 387 µmol m^−2^ s^−1^; LL: 107 µmol m^−2^ s^−1^. *, **, *** significant differences compared to the control plants on the same day at the *p* < 0.05, 0.01 and 0.001 levels, respectively. **^#^**, **^##^** significant differences compared to the GL plants on the same day at the *p* < 0.05, and 0.01 levels, respectively. (**A**: free oHCA in the leaves; **B**: free oHCA in the roots).

The level of ortho-hydroxycinnamic acid (oHCA), a putative pre-cursor of SA, also increased during acclimation and chilling in the leaves and dropped back to the initial level during recovery (Figure 2A). A much higher increase was detected in non-acclimated plants at 5 °C and on the first day of recovery, but it also dropped later. No significant changes were detected in the roots, but the oHCA level was twice as high than in the leaves (Figure 2B).

**Figure 3:**
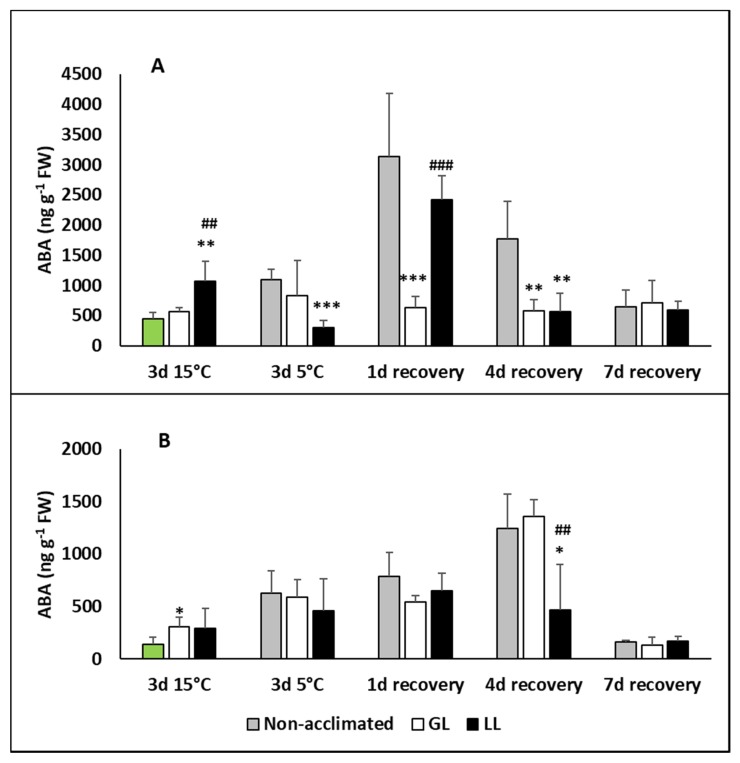
Changes in the abscisic acid (ABA) contents during cold acclimation (15/13 °C), chilling (5 °C) and recovery in the leaves and roots of young maize plans: control plants (22/20 °C, 387 µmol m^−2^ s^−1^). Light intensities during hardening: GL: 387 µmol m^−2^ s^−1^; LL: 107 µmol m^−2^ s^−1^. *, **, *** significant differences compared to the control plants on the same day at the *p* < 0.05, 0.01 and 0.001 levels, respectively. **^##^**, **^###^** significant differences compared to the GL plants on the same day at the *p* < 0.05 and 0.001 levels, respectively. (**A**: free ABA in the leaves; **B**: free ABA in the roots).

The level of abscisic acid (ABA) did not change during the acclimation in the leaves of GL plants and it remained at the same level during the chilling and recovery period (Figure 3A). A slight increase could be detected in LL plants during the acclimation, but it was much more pronounced after 1d recovery. The ABA level of non-acclimated plants increased only during the recovery, especially on the first day, but it returned to the initial level in all the treatments on the seventh day. No changes were measured during acclimation in the roots (Figure 3B). Although the chilling increased the ABA level, no difference could be observed between the acclimated and non-acclimated plants. A slight increase in ABA level was determined in non-acclimated and GL plants during recovery, which dropped back at the end of recovery.

**Figure 4:**
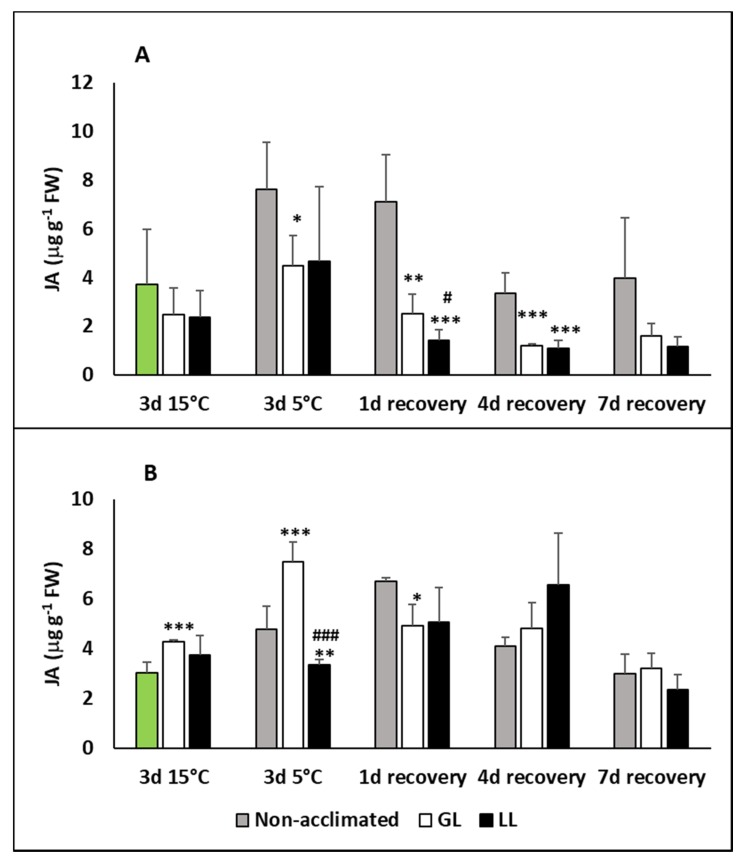
Changes in the jasmonic acid (JA) contents during cold acclimation (15/13 °C), chilling (5 °C) and recovery in the leaves and roots of young maize plans: control plants (22/20 °C, 387 µmol m^−2^ s^−1^). Light intensities during hardening: GL: 387 µmol m^−2^ s^−1^; LL: 107 µmol m^−2^ s^−1^. *, **, *** significant differences compared to the control plants on the same day at the *p* < 0.05, 0.01 and 0.001 levels, respectively. **^#^**, **^###^** significant differences compared to the GL plants on the same day at the *p* < 0.05 and 0.001 levels, respectively. (**A**: free JA in the leaves; **B**: free JA in the roots).

The jasmonic acid (JA) level did not change in the leaves during acclimation, but chilling significantly increased it in the cold-acclimated GL plants and especially in the non-acclimated ones (Figure 4A). On the first day of recovery, the JA level dropped back to the initial level only in the acclimated plants, but from the fourth day its level was sometimes even less than before the acclimation period. No effect of the light could be observed on the amount of JA during the acclimation, chilling and recovery periods. On the contrary, a slight elevation could be seen in the roots acclimated under GL conditions, but no changes in the LL plants (Figure 4B). The JA level in the roots increased during the chilling in non-acclimated plants, and more pronouncedly in GL, while no changes could be seen in LL plants. Interestingly, some rise could be observed in non-acclimated and LL plants on the first day of recovery, but a decrease in GL roots. On the seventh day of recovery, the JA level was similar to the initial one.

### 2.2. Oxidative Stress and Antioxidants

**Figure 5:**
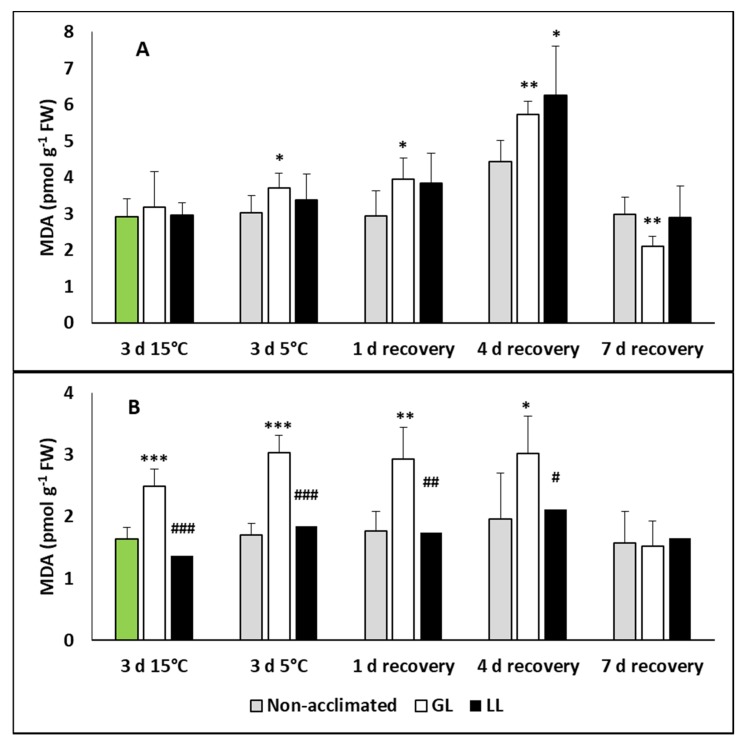
Changes in the malondialdehyde (MDA) contents during cold acclimation (15/13 °C), chilling (5 °C) and recovery in the leaves and roots of young maize plans: control plants (22/20 °C, 387 µmol m^−2^ s^−1^). Light intensities during hardening: GL: 387 µmol m^−2^ s^−1^; LL: 107 µmol m^−2^ s^−1^. *, **, *** significant differences compared to the control plants on the same day at the *p* < 0.05, 0.01 and 0.001 levels, respectively. **^#^**, **^##^**, **^###^** significant differences compared to the GL plants on the same day at the *p* < 0.05, 0.01 and 0.001 levels, respectively. (**A**: leaves; **B**: roots).

For detection of the oxidative stress, the malondialdehyde (MDA) level was measured in the leaves and roots of plants during acclimation, chilling and recovery. No changes were detected in the leaves during the acclimation and chilling periods, and only a slight increase was detected in the leaves of hardened plants on the fourth day of recovery (Figure 5A). However, the MDA level increased significantly in the roots of GL plants (Figure 5B).

**Figure 6:**
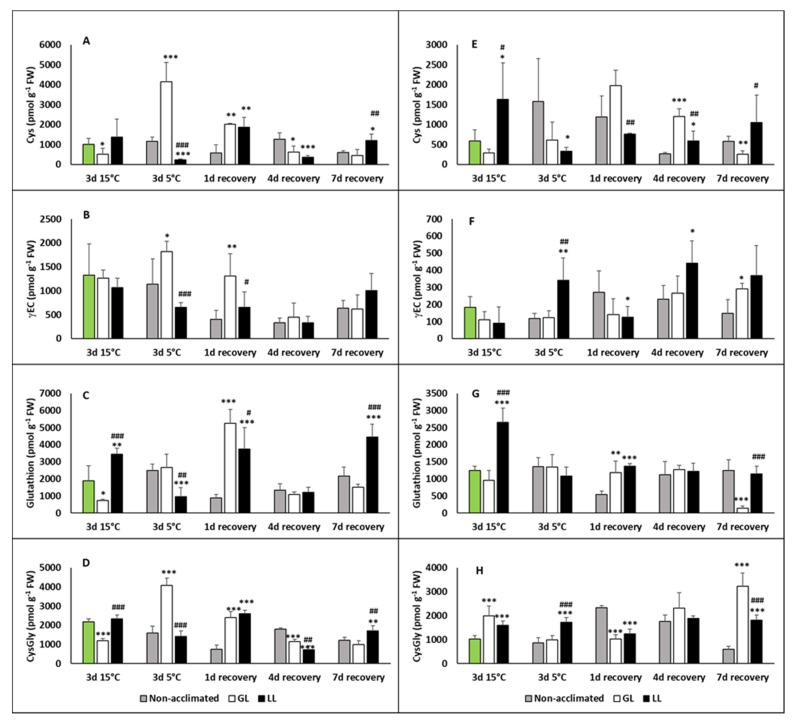
Changes in the thiol contents during cold acclimation (15/13 °C), chilling (5 °C) and recovery in the leaves and roots of young maize plants. Control plants (22/20 °C, 387 µmol m^−2^ s^−1^). Light intensities during hardening: GL: 387 µmol m^−2^ s^−1^; LL: 107 µmol m^−2^ s^−1^. *, **, *** significant differences compared to the control plants on the same day at the *p* < 0.05, 0.01 and 0.001 levels, respectively. **^#^**, **^##^**, **^###^** significant differences compared to the GL plants on the same day at the *p* < 0.05, 0.01 and 0.001 levels, respectively. (**A**: cysteine in the leaves; **B**: γ-glutamyl-cysteine in the leaves; **C**: gluthathione in the leaves; **D**: cysteinyl–glycine in the leaves; **E**: cysteine in the roots; **F**: γ-glutamyl-cysteine in the roots; **G**: gluthathione in the roots; **H**: cysteinyl–glycine in the roots).

Thiols, especially glutathione (γ-L-glutamyl-L-cysteinyl–glycine (GSH)), have an important role to play in the defence processes against oxidative stress. Thus, thiol compounds, namely GSH and its precursors, cysteine (Cys) and γ-L-glutamyl-L-cysteine (γEC) were also analysed. Cys levels increased after chilling in the leaves of GL plants and was still higher on the first day of recovery (Figure 6A). Similar tendencies could be seen in the amount of γEC (Figure 6B). Compared to these, the GSH level increased in LL plants after acclimation and decreased during the chilling (Figure 6C), and a dramatic rise was observed on the first day of recovery, both in GL and LL plants. Cysteinyl–glycine (CysGly), a degradation product of GSH, had the highest amount after the chilling in GL plants, but dropped back to the initial level during the recovery (Figure 6D). Cys increased after acclimation in the roots of LL plants, but after chilling it was at the initial level (Figure 6E). An increased level of it was detected in non-acclimated plants after chilling and it was still high on the first day of recovery. The GL plants had elevated levels of root Cys on the first and fourth days of recovery, while root γEC increased only in LL plants after chilling and it was also higher on the fourth and seventh days of recovery (Figure 6F). Elevated levels could be also seen in the non-acclimated and GL plants during recovery, but this level was still lower than in LL plants. A big enlargement in the GSH amount was measured in LL plants after acclimation, but after the chilling, and during recovery, the initial level was detected in the roots of the plants (Figure 6G). The amount of CysGly increased during acclimation and remained at the same level in LL plants after chilling and during the recovery (Figure 6H). It dropped to the initial level in GL plants after chilling, but it started to increase during the recovery, and the highest level was detected after seven days.

**Figure 7:**
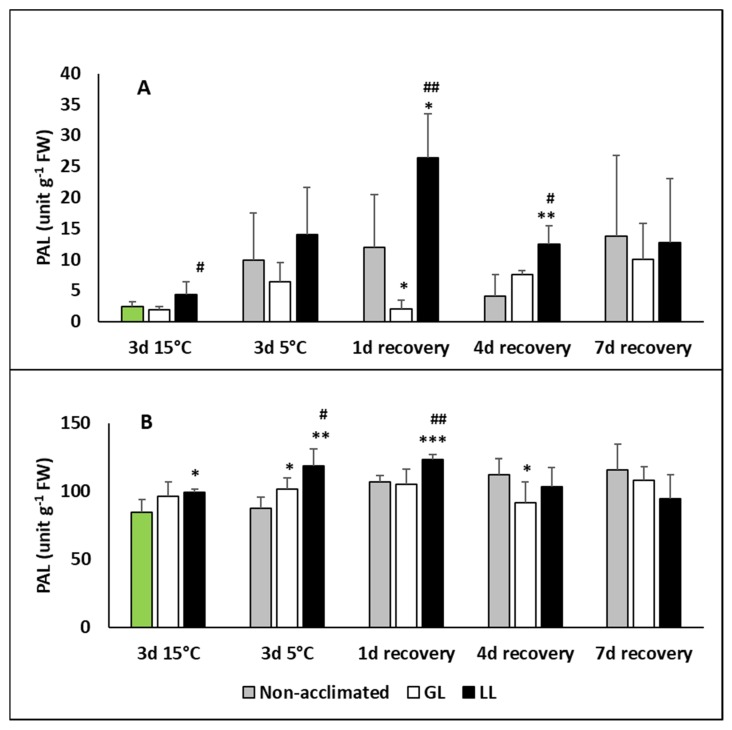
Changes in the phenylalanine ammonia lyase (PAL) contents during cold acclimation (15/13 °C), chilling (5 °C) and recovery in the leaves and roots of young maize plans: control plants (22/20 °C, 387 µmol m^−2^ s^−1^). Light intensities during hardening: GL: 387 µmol m^−2^ s^−1^; LL: 107 µmol m^−2^ s^−1^. *, **, *** significant differences compared to the control plants on the same day at the *p* < 0.05, 0.01 and 0.001 levels, respectively. **^#^**, **^##^** significant differences compared to the GL plants on the same day at the *p* < 0.05 and 0.01 levels, respectively. (**A**: leaves; **B**: roots).

PAL is a key enzyme of the phenylpropanoid metabolism. It has a role either in the SA or flavonol/anthocyanin biosynthesis. The PAL activity increased in the leaves of LL plants after acclimation, but showed a much higher amount on the first day of recovery (Figure 7A). Its activity was higher in the roots than in the leaves and an increase could be seen after acclimation, chilling and 1 d recovery, mainly in LL plants (Figure 7B).

Flavonols (kaempferol (K), quercetin (Q), myricetin (M) and rutin (R)) and anthocyanins were also analysed. K, Q and M were in a much lower amount (0.1–2 µg g^−1^ FW) both in the leaves and roots than rutin (100–130 µg g^−1^ FW). Only the R level increased in the leaves, especially in LL plants, but after 4 d recovery it was lower than the initial level (Table 1). There were no substantial changes in the roots, only the M increased at 3 d and at 15 °C in GL and LL plants (Table 1). 

**Figure 8:**
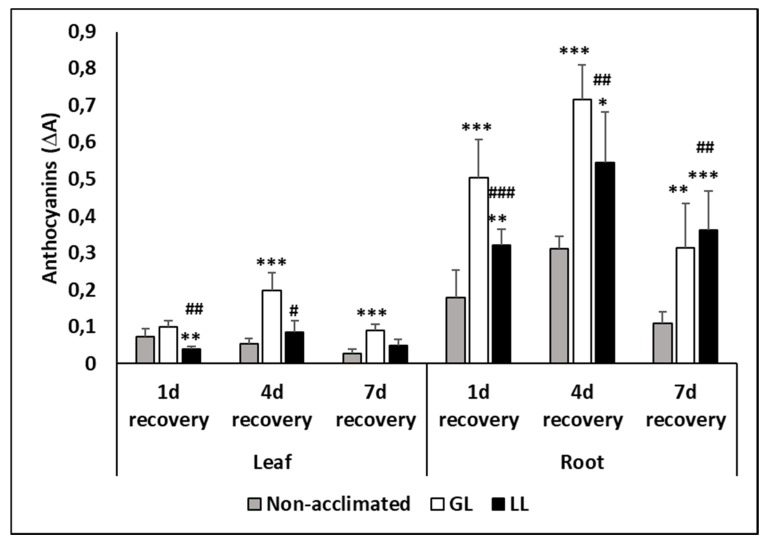
Changes in the anthocyanin contents during cold acclimation (15/13 °C), chilling (5 °C) and recovery in the leaves and roots of young maize plants. Light intensities during hardening: GL: 387 µmol m^−2^ s^−1^; LL: 107 µmol m^−2^ s^−1^. *, **, *** significant differences compared to the control plants on the same day at the *p* < 0.05, 0.01 and 0.001 levels, respectively. **^#^**, **^##^**, **^###^** significant differences compared to the GL plants on the same day at the *p* < 0.05, 0.01 and 0.001 levels, respectively.

Anthocyanins could only be detected during the recovery period. It accumulated mainly in the roots of GL plants but changes in the leaves were also observed (Figure 8). The highest amount was detected on the fourth day of recovery, but it was still high on the seventh day in the acclimated plants.

### 2.3. Gene Expression Studies

Based on a microarray assay, the changes in the gene expression levels during the cold acclimation period under various light conditions have been analysed in our previous study [12]. In the present work—based on the same microarray database—gene expressions related to the above characterised changes in plant hormone metabolisms (Section 2.1.) and antioxidant capacity (Section 2.2.) have been further analysed in the leaves at the end of acclimation period.

A gene encoding a PAL enzyme was downregulated and genes encoding the enzyme related to the SA-MeSA (methyl-SA) conversation (salicylate carboxymethyltransferase), and enzymes related to the synthesis of ABA (zeaxanthin epoxidase (ZEP)) and JA (12-oxo-phytodienoic acid reductase7) and phenypropanoid metabolism (cinnamyl-alcohol dehydrogenase) were upregulated in GL compared to the control plants (Table S1). Chorismate mutase 1 (related to the SA biosynthesis as a part of the shikimic acid pathway) and violaxanthin de-epoxidase were downregulated, while enzymes of ABA (aldehyde oxidase, ZEP) and JA (12-oxo-phytodienoic acid reductase7, allene oxide synthase 1) biosynthesis were upregulated in LL compared to the control plants. In a comparison of GL and LL plants, salicylate carboxymethyltransferase, aldehyde oxidase, allene oxide synthase 1 were upregulated in GL plants, while chorismate mutase 1 and violaxanthin de-epoxidase were downregulated.

Gene expression levels related to the antioxidants were also examined at the end of the acclimation period in the leaves of the plants. Glutathione-S-transferase (GST) was downregulated and ascorbate peroxidases were upregulated in GL plants compared to the control (Table S2). Mainly the enzymes of the ascorbate–glutathione cycle (glutathione reductase, monodehydroascorbate reductase 5, dehydroascorbate reductase) were downregulated and GSTs were upregulated in LL compared to the control. Enzymes related to the flavonol/anthocyanin biosynthesis (isoflavone reductase, dihydroflavonol-4-reductase) were upregulated in GL and LL plants compared to the control (Table S3). In a comparison of GL–LL plants, GST and flavonol/anthocynin biosynthesis enzymes were upregulated and some enzymes of the ascorbate (ASC)–GSH cycle (glutathione reductase, monodehydroascorbate reductase 5) were downregulated in GL compared to the LL plants.

## 3. Discussion

Growing plants at suboptimal but non-lethal temperatures may help them to prepare for a subsequent, more severe cold stress. In the case of hardy winter cereals, it may also lead to development of frost tolerance, which enables plants to survive low temperatures well below 0 °C. Light during the hardening period is also essential for the development of adequate freezing tolerance. Its role and mode of action have been widely studied [6,20,21]. But the role of light during the cold acclimation period in the case of chilling sensitive plants is hardly documented. When light is accompanied by low growth temperature, usually, photoinhibitory damage can be detected, which may substantially contribute to the chilling injury in cold-sensitive plants. However, our previous study [12] indicated that, in spite of its photoinhibitory effects, light is also essential for the high level of cold acclimation in young maize plants. In order to better understand the molecular mechanisms of the light-related cold acclimation processes, in recent works, certain stress-related plant hormones, focusing mainly on SA and its related compounds, were also followed during the cold acclimation, chilling, and a subsequent recovery periods. SA is also a Janus-faced compound: its exogenous application provided protection against chilling injury in young maize plants [22], but its relatively high endogenous level inhibited the growth of *Arabidopsis* plants at low temperatures [23]. In the present work, the main changes could be observed in the free SA levels in the leaves, and it was lower in acclimated plants than in the controls. Although, the gene expression level of PAL enzyme was downregulated in GL plants under cold conditions, the PAL enzyme activity did not show remarkable differences, which could have been the reason for the lower SA contents in the leaves. However, at the end of the hardening period, the gene expression of the salicylate caboxymethyltransferase, an enzyme responsible for SA-MeSA conversation, increased in the leaves of GL plants both under cold and control conditions, suggesting the dominant effect of light, rather than the cold MeSA, is a volatile form of SA and is part of the SA signalling pathway [24] and the decreased level of free SA could at least be partly a result of this SA-MeSA conversation. Interestingly, parallel to this, the expression of chorismate mutase 1 was also downregulated in GL plants, suggesting that the branch point of the shikimate pathway to channel amino acid precursors to the biosynthesis of phenylalanine and tyrosine and away from that of tryptophan was lower at GL [25].

ABA is a stress hormone, playing a role in the responses to various environmental stimuli [26]. In our experiment, the level of ABA did not correlate with the level of tolerance. Its level mainly increased in the leaves during the post-chilling recovery period. It seems that it is mainly related to the level of the stress, and less related to the level of the acclimation. At the end of acclimation, the synthesis gene of ABA (ZEP) was upregulated both in GL and LL plant compared to the non-acclimated plants, but there was no difference between the GL and LL plants. In addition, the expression of violaxanthin de-epoxidase, which is responsible for the catalysation of the reaction in the opposite direction, was downregulated in LL compared to the control under cold conditions, while it was upregulated in the GL compared to the LL plants. It has been found that the gene expression of ZEP showed mainly circadian rhythms in the leaves rather than changes in the daily periods of stress, and no changes were detected in the amount of the enzyme [27]. On the other hand, light was required for the gene expression of ZEP, because cold treatment only induced the genes of the ABA biosynthesis in light, as was demonstrated in *Arabidopsis* plants [28].

A similar trend could be observed in the JA levels, which mainly increased in the leaves of non-acclimated plants. In addition, at the end of acclimation, the gene of JA biosynthesis (12-oxo-phytodienoic acid reductase7) was upregulated both in GL and LL plants compared to the non-acclimated plants, but there was no difference between GL and LL plants, while another gene encoding allene oxide synthase 1 was shown to exhibit higher upregulation in GL plants. Chilling temperatures may increase the generation of the reactive oxygen species (ROS), especially in light. Plants generally respond to such conditions by activating antioxidant systems. oHCA, a precursor of SA, belongs to the hydroxycinnamic acid family, and it also has antioxidant properties. Although the oHCA level was lower in the acclimated plants, and no pronounced differences were observed between the GL and LL plants, the gene of cinnamyl-alcohol dehydrogenase, related to the cinnamic acid metabolism, was upregulated in GL plants. This latter result is in accordance with earlier findings, where two genes playing roles in the synthesis of hydroxycinnamic acids were also upregulated at a low temperature in light in *Arabidopsis* plants [28]. Parallel to the induction of the gene, a cold-induced increase in the oHCA level was also detected in young maize plants. However, this increase also occurred in the dark. This response was entirely different from that which was earlier found during the cold hardening period in winter wheat plants, where a substantial increase in the oHCA level only occurred in light, but not in the dark [29]. However, the pattern of leaf oHCA content under the recovery period showed similar differences and changes, as was found for the SA. It seems that the exact role of oHCA as a precursor and/or antioxidant compound during the cold acclimation period still requires intensive further research.

Hydroxycinnamic acid biosynthesis, like SA metabolism, is part of the phenylpropanoid pathway which involves the synthesis of various antioxidants, such as flavonols, anthocyanins, etc., and one of the key enzymes of this pathway is PAL. It was found earlier that light had a role not only in the development of chilling injury, but also in the appearance of the post-chilling symptoms in young maize plants [30]. Furthermore, light was also required for the synthesis of anthocyanins when plants were transferred from chilling conditions to optimum growth temperatures [31]. In the present work, although the recovery was in light, and only the acclimation was carried out at different illuminations, the differences in anthocyanin level could be seen both in the leaves and roots. Plants acclimated at low light had much lower anthocyanin content compared to the GL plants. Interestingly, differences in the anthocyanin levels did not follow the changes in the activity of the PAL enzyme. Furthermore, the changes in the various flavonols, such as K, Q, M or R, either in the leaves or in the roots, were more temperature- than light-dependent. This was in accordance with the gene expression changes, as the transcript level of the genes related to flavonoid metabolism was also upregulated in GL and LL plants under cold conditions.

GSH also acts as an antioxidant by quenching ROS. The ASC–GSH cycle plays an important role in this respect. In *Arabidopsis*, cold treatment in light induced the expression of genes of two GSH-dependent peroxidases, while the genes of the ASC–GSH cycle were not induced [28]. In our case similar changes were found in maize plants, the genes of the ASC–GSH cycle enzymes were downregulated, while genes encoding GST were upregulated in GL plants compared to the LL conditions during cold acclimation. However, the accumulation of GSH was also light dependent. The synthesis of GSH is a two-step reaction: the first step is the formation of γEC from Cys catalysed by γEC synthetase then the GSH synthetase catalyses the addition of a glycine. The rate-limiting step is the γEC synthesis [32]. In a recent study, the highest GSH level was found at optimum light conditions in wheat plants. Either the higher or lower illumination caused lower GSH accumulation [33]. In the present work, the highest GSH level was found in the leaves of GL plants on the first day of recovery, but the pre-cursors of GSH (Cys, γEC) started to accumulate during chilling in GL plants. However, this accumulation did not cause an increase in the GSH level, because the enhancement of the degradation processes led to high levels of CysGly. In contrast to the GL plants, under low light conditions, higher GSH accumulation occurred during the acclimation period.

GSH is not only an antioxidant, it can also interact with plant hormones. GSH regulates SA accumulation via the expression of genes of isochorismate synthetase 1 in *Arabidopsis* plants and it can lead to an increase in the intracellular hydrogen peroxide level for the activation of SA signalling [34]. In our case, GSH accumulation was not accompanied by SA accumulation either in the free or bound forms. In another study, the GSH content was higher after SA treatment in maize plants, while it was lower after the application of ABA [35]. In the present work, it was also found that a higher GSH content was coupled with a lower ABA level. However, under the present conditions, the antagonistic relationship between SA and ABA, or between SA and JA, could not be detected.

## 4. Materials and Methods

### 4.1. Plant Material and Growth Conditions

Maize plants were grown for 11 days at 22/20 °C at higher photosynthetic photon flux density (PPFD), (growth light, GL = 387 µmol m^−2^ s^−1^) with 16/8 h light/dark periods. Some of the plants were then cold acclimated at 15/13 °C for 3 days, either at GL or at moderate low light intensity (LL) 107 µmol m^−2^ s^−1^. Afterwards, all plants were transferred to 5 °C at a continuous growth light (GL) for 3 days, followed by a seven-day recovery period at 22 °C under GL conditions. Samples were collected for biochemical analysis from the control plants, and from treated plants after the acclimation (GL and LL plants), chilling and during recovery periods (non-acclimated, GL and LL plants).

All the chemicals were purchased from Sigma-Aldrich or Merck (Merck Group, Darmstadt, Germany).

### 4.2. Estimation of Lipid Peroxidation

The lipid peroxidation analysis was based on the measurement of the MDA level according to Gondor et al. [36] using 0.5 g plant material. Leaves and roots were extracted with 1.5 mL 0.1% (*w*/*v*) tricholoracetic acid and, after incubation with 0.5% (*w*/*v*) thiobarbituric acid at 90 °C for 30 min, the MDA equivalent compounds were measured spectrophotometrically at 532 nm, with the subtraction of non-specific absorption at 600 nm. MDA was then quantified using an extinction coefficient of 155 mM^−1^·cm^−1^ and expressed as nM·g^−1^ fresh weight. Five replicates were measured from each treatment and at least three leaves were used for one replicate.

### 4.3. Extraction and Analytical Procedure of Salicylic Acid and Flavonols

Flavonoids, JA, ABA, SA and its precursors were measured according to Meuwly and Métraux [37] and Pál et al. [38] using 1 g plant material. The leaves and the roots were ground in liquid nitrogen in a mortar and pestle, in the presence of 0.5 g quartz sand. The tissue powder was transferred to a centrifugation tube and mixed with 2 mL of 70% methanol. The extract was centrifuged at 10,000× *g* for 20 min. The pellet was resuspended in 2 mL of 90% methanol, vortexed and centrifuged as above. The methanol content was evaporated from the mixed supernatants at room temperature under a vacuum. One millilitre of 5% (*w*/*v*) trichloroacetic acid was added to the residual aqueous phase, and the mixture was centrifuged at 15,000× *g* for 10 min. The supernatant was gently partitioned against 3.6 mL of a 1:1 (*v*/*v*) mixture of ethyl acetate/cyclohexane. The upper organic layers contained the free phenolic portion. The aqueous phases containing the methanol-soluble bound phenolics were acid-hydrolysed. 1.3 mL of 8 N HCl was added to the aqueous phase and incubated for 60 min at 80 °C, before partitioning as above. The organic phases were evaporated to dryness under a vacuum and stored at −20 °C. Just prior to the HPLC analysis, the evaporated samples were resuspended in 0.5 mL 15% acetonitrile and filtered through a 0.45 m pore size polytetrafluoroethylene (PTFE) membrane filter (Millipore, Merck Group, Darmstadt, Germany). The detailed description of the HPLC analysis was described by Janda et al. [39].

### 4.4. Measurement of Thiols

A total of 200 mg plant material was extracted with 1 mL of 0.1 M HCl and centrifuged for 20 min with 10,000 rpm. Total thiol content was determined after the supernatant was reduced (120 µL) with dithiothreitol, and derivatization was carried out with monobromobimane [40]. The samples were analysed after the separation of Cys, γEC, CysGly (a degradation product of GSH) and GSH by reverse-phase HPLC (W996, Waters, Milford, MA, USA) using a scanning fluorescence detector (W2474, Waters, Milford, MA, USA). The detailed parameters of the analysis were described by Janda et al. [39].

### 4.5. Determination of Phenylalanine Ammonia Lyase (PAL) Activity

PAL activity was measured according to Gao et al. [41] using 1 g leaves and roots homogenised with 4 mL 50 mM TRIS-HCl buffer (pH 8.8 containing 5 mM β–mercaptoethanol and 4% (*v*/*v*) polyvinylpyrrolidone). After centrifugation (10 min 10,000× *g*), 250 µL supernatant was used for enzyme activity determination in a 3 mL total volume of reaction mixture containing 50 mM phosphate buffer (pH 8.8) and 50 mM phenylalanine. The reaction was stopped with 10% trichloroacetic acid after a one-hour incubation at 37 °C and the produced *trans*-cinnamic acid was detected at 290 nm spectrophotometrically and expressed as enzyme units per g fresh weight (U g^−1^ FW).

### 4.6. Determination of Anthocyanin Content

The method described by Kho et al. [42] was used for the anthocyanin content determination using 0.5 g plant tissue extracting with 2 × 300 µL of methanol containing 1% HCl and was kept at 4 °C for overnight. After removing the chlorophylls with 500 µL chloroform and centrifugation at 10,000× *g* for 30 min, the total anthocyanin content was determined from the supernatant at 530 nm spectrophotometrically.

### 4.7. Statistical Analysis

The experiments were repeated three times and representative data are shown. The results were the means of five measurements. The data were statistically evaluated using the standard deviation, ANOVA and *t*-test methods.

## 5. Conclusions

**Figure 9:**
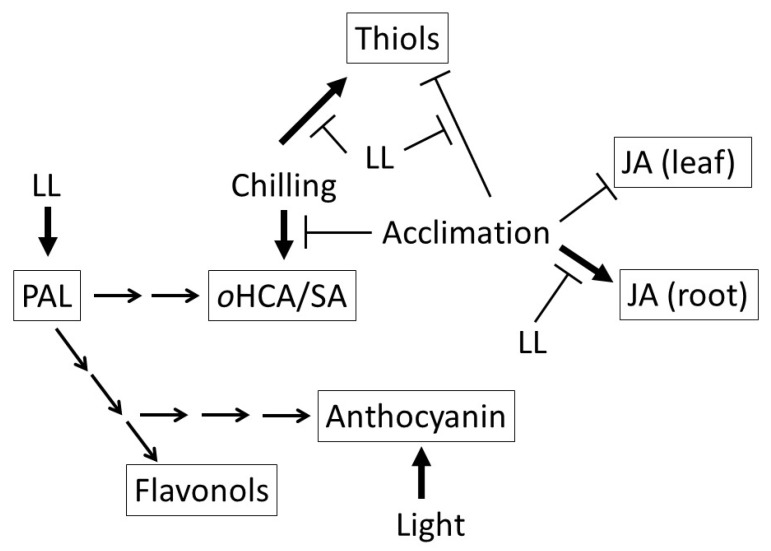
Schematic representation of the effect of the light on phenolic metabolism in young maize plants during cold acclimation. JA: jasmonic acid; LL: low light intensity (107 µmol m^−2^ s^−1^) during cold acclimation; ortho-hydroxycinnamic acid (oHCA); phenylalanine ammonia lyase (PAL).

Different light conditions during the cold acclimation period differentially affected certain stress-related mechanisms in young maize plants (Figure 9). Interestingly, changes were also light dependent in the root, not only in the leaves. PAL has a key role in the synthesis of various secondary metabolic routes, including compounds with antioxidant activities, and was mainly activated under limited light conditions. However, light-induced anthocyanin accumulation occurred not only in the leaves, but in the roots, too. Chilling induced the accumulation of SA and one of its putative precursors, oHCA. However, this accumulation was moderate in the cold-acclimated plants. Low temperature reduced the accumulation of JA in the leaves, but induced it in the roots. The highest GSH level was found on the first day of the recovery period in the leaves of GL plants, but its pre-cursors started to accumulate even during the chilling. The level of ABA is mainly related to the level of stress, and less related to the level of the acclimation. According to these results, it was demonstrated that LL condition partly modifies the acclimation processes after only 1 day at the gene expression level, which, in turn, may result in hormonal and metabolic shifts, sometimes in different ways, in the leaves and roots of the plants.

## Figures and Tables

**Table 1:** Changes in the flavonol contents during cold acclimation (15/13 °C), chilling (5 °C) and recovery in the leaves and roots of young maize plants. Control: 22/20 °C, 387 µmol m^−2^ s^−1^. NA: non-acclimated plants. Light intensities during hardening: GL: 387 µmol m^−2^ s^−1^; LL: 107 µmol m^−2^ s^−1^. *, **, *** significant differences compared to the control plants on the same day at the *p* < 0.05, 0.01 and 0.001 levels, respectively. **^#^**, **^##^**, **^###^** significant differences compared to the GL plants on the same day at the *p* < 0.05, 0.01 and 0.001 levels, respectively.

**Leaf**	**3 d 15 °C**			**3 d 5 °C**			**1 d recovery**			**4 d recovery**			**7 d recovery**		
	**Control**	**GL**	**LL**	**NA**	**GL**	**LL**	**NA**	**GL**	**LL**	**NA**	**GL**	**LL**	**NA**	**GL**	**LL**
Kaempferol	0.76 ± 0.11	1.04 ± 0.09 **	0.9 ± 0.07 *^#^	0.92 ± 0.04	0.96 ± 0.16*^##^	0.725 ± 0.1	1.1 ± 0.46	0.96 ± 0.15	0.92 ± 0.11	2.125 ± 0.44	1.04 ± 0.09 ***	0.96 ± 0.18 ***	1.4 ± 0.34	0.74 ± 0.05 **	0.72 ± 0.08 **
Quercetin	1.43 ± 0.1	1.52 ± 0.38	1.52 ± 0.16	1.76 ± 0.15	1.52 ± 0.23	1.625 ± 0.17	1.66 ± 0.4	1.4 ± 0.12	1.28 ± 0.13	1.42 ± 0.08	1.25 ± 0.06	1.36 ± 0.29	1.12 ± 0.04	1.02 ± 0.11	0.94 ± 0.15 *
Myricetin	0.31 ± 0.07	0.59 ± 0.33	0.71 ± 0.42	0.24 ± 0.05	0.24 ± 0.09	0.19 ± 0.22	nd	0.58 ± 0.21 ***	0.65 ± 0.35 **	nd	nd	nd	nd	0.43 ± 0.12 ***	0.3 ± 0.2 *
Rutin	83.28 ± 10.9	101.38 ± 8.3 **	104.38 ± 7.77 **	87.4 ± 10.57	97.12 ± 18.51	121.83 ± 3.38 ***^#^	89.78 ± 6.44	82.12 ± 0.82 *	96.68 ± 5.1 ^###^	41.98 ± 15.44	31.9 ± 8.24	50.28 ± 8.63 ^#^	37.52 ± 12.59	38.54 ± 6.79	37.12 ± 3.07
**Root**	**3 d 15 °C**			**3 d 5 °C**			**1 d recovery**			**4 d recovery**			**7 d recovery**		
	**Control**	**GL**	**LL**	**NA**	**GL**	**LL**	**NA**	**GL**	**LL**	**NA**	**GL**	**LL**	**NA**	**GL**	**LL**
Kaempferol	0.04 ± 0.02	0.05 ± 0.01	0.06 ± 0.02	0.13 ± 0.03	0.13 ± 0.01 *^##^	0.09 ± 0.02	0.19 ± 0.03	0.16 ± 0.03	0.14 ± 0.03	0.21 ± 0.03	0.25 ± 0.05	0.15 ± 0.09	0.06 ± 0.02	0.04 ± 0.02	0.07 ± 0.04
Quercetin	1.18 ± 0.14	1.36 ± 0.06 *	1.38 ± 0.1 *	1.36 ± 0.26	1.44 ± 0.27	1.18 ± 0.16	1.45 ± 0.17	1.02 ± 0.13 **	1.26 ± 0.13^#^	1.14 ± 0.11	1.2 ± 0.19	0.93 ± 0.05**^#^	0.8 ± 0.1	0.9 ± 0.19	1.08 ± 0.08 **
Myricetin	8.02 ± 0.92	26.52 ± 8.99 **	29.34 ± 13.61 **	11.28 ± 6.04	7.76 ± 2.38	8.68 ± 6.07	4.5 ± 4.6	2 ± 1.57	2.84 ± 1.8	3.28 ± 1.15	6.58 ± 2.6*	7.72 ± 8.01	7.94 ± 4.25	7.72 ± 1.87	11.04 ± 2.9
Rutin	132.55 ± 18.47	130.86 ± 13.14	118.8 ± 8.76	110.1 ± 6.81	105.16 ± 8.88	97.18 ± 15.11	104.75 ± 2.06	97.68 ± 3.48 *	95.02 ± 6.85	106.56 ± 8.13	100.44 ± 3.23	117.17 ± 20.45	126.12 ± 4.68	119.36 ± 13.55	107.8 ± 12.77 *

## Supplementary Materials

Supplementary Materials can be found at https://www.mdpi.com/1422-0067/21/6/1942/s1. Table S1: BLASTX search hits of cold- and light-induced significantly differentially expressed transcripts related to hormone biosynthesis. Table S2: BLASTX search hits of cold- and light-induced significantly differentially expressed transcripts related to thiol metabolism. Table S3: BLASTX search hits of cold- and light-induced significantly differentially expressed transcripts related to flavonoid metabolism.

## Abbreviations

ABA	abscisic acid
ASC	ascorbate
CysGly	cysteinyl–glycine
GL	growth light, 387 µmol m^−2^ s^−1^
GSH	gluthathione (γ-L-glutamyl-L-cysteinyl–glycine)
GST	glutathione-S-transferase
JA	jasmonic acid
K	kaempferol
LL	low light intensity, 107 µmol m^−2^ s^−1^
MDA	malondialdehyde
M	myricetin
oHCA	ortho-hydroxycinnamic acid
PAL	phenylalanine ammonia lyase
Q	quercetin
R	rutin
SA	salicylic acid
ZEP	zeaxanthin epoxidase
γEC	γ-L-glutamyl-L-cysteine
